# Urologie in der Oberpfalz – Was uns Archäologie, historische Forschung und Geschichtsschreibung verraten: Teil 1: von römischer Antike bis Spätmittelalter

**DOI:** 10.1007/s00120-024-02296-5

**Published:** 2024-02-28

**Authors:** W. Otto

**Affiliations:** 1 Lehrstuhl für Urologie, Universitätsklinikum Regensburg, Franz-Josef-Strauß-Allee 11, 93053 Regensburg, Deutschland; 2 UROLOGIE im GesundheitsFORUM, Paracelsusstraße 2, 93053 Regensburg, Deutschland; 3 Arbeitskreis Geschichte der Urologie der Deutschen Gesellschaft für Urologie, Uerdinger Str. 64, 40474 Düsseldorf, Deutschland

**Keywords:** Urologie, Römisches Reich, Castra regina, Regensburg, Bayern, Oberpfalz, Spitalwesen, Urology, Roman Empire, Castra Regina, Regensburg, Bavaria, Upper Palatinate, Early hospital systems

## Abstract

Seit dem Jahr 1924 gibt es in Deutschland die Möglichkeit, eine Facharztanerkennung für Urologie zu erwerben. Bereits im ausgehenden Kaiserreich und der Weimarer Republik entstanden erste Klinikabteilungen zur Behandlung urologischer Krankheitsbilder. Der Beginn fachärztlicher Versorgung im bayerischen Regierungsbezirk Oberpfalz, in Teilen bis in die zweite Hälfte des vergangenen Jahrhunderts hinein als „Armenhaus“ Deutschlands bekannt, liegt aber deutlich später. Dennoch lässt sich hier die Linie „urologischer“ Tätigkeit in sehr viel weiter zurückliegende Epochen zurückverfolgen. Dabei muss es als naturgemäß hingenommen werden, dass die Nachweislage in längst vergangenen Zeiten schwach ausgeprägt ist. Zumal bei einem Thema wie der Krankenbehandlung und Gesundheitserhaltung, das uns Heutigen nicht nur als Wissenschaft, sondern auch ganz individuell oft als zentraler Lebensschwerpunkt gilt, in der Geschichte aber oft genug nur in der Seuchenbekämpfung und in anekdotischen Beschreibungen ihren Ausdruck fand. Ein Umstand, der beim Blick in die Geschichte der Urologie freilich die ein oder andere interessante Quelle erhoffen lässt. So möchte der vorliegende Beitrag nicht nur die Entwicklungsgeschichte der Urologie in zeithistorischer Prägung bis in unsere Tage in einem ländlich geprägten Raum nachzeichnen, sondern auch auf die Behandlung urologischer Krankheitsbilder in diesem Gebiet seit der Spätantike eingehen – und dazu einladen, an anderen Erinnerungsorten der Urologie ähnlich zu verfahren.

## Abstract (en)

Since 1924, it has been possible to qualify as a specialist in urology in Germany. The first hospital departments for the treatment of urological conditions were established as early as the end of the German Empire and the Weimar Republic. However, the beginning of specialist medical care in the Bavarian administrative district of Upper Palatinate, often known as the “poorhouse” of Germany until the second half of the last century, was much later. Nevertheless, the lineage of “urological” activity here can be traced back to much earlier eras. Of course, it must be accepted that the evidence from times long past is weak. This is especially applicable when it comes to a topic such as the treatment of the sick and the preservation of health, which is not only considered a science but also often a central focus of life for us today, because in history it was often only mentioned in the fight against epidemics and in anecdotal descriptions. This is a circumstance which, when looking at the history of urology, gives rise to the hope of finding one or two interesting sources. This article not only aims to trace the history of the development of urology in a rural area from contemporary history to the present day, but also to explore the treatment of urological diseases in this region since late antiquity. Furthermore, we invite readers to look at other places of urological remembrance in a similar way.

Zunächst sei aber erst einmal für die Leserschaft dargelegt, was die „Oberpfalz“ eigentlich ist und wie sich diese – zum besseren Verständnis der folgenden medizinhistorischen Ausführungen und des Status quo – über die Jahrhunderte hinweg entwickelt hat. Diese Informationen können und sollen an dieser Stelle natürlich nur kursorisch sein.

## Topographie und allgemeine historische Vorbemerkungen

**Abb. 1:**
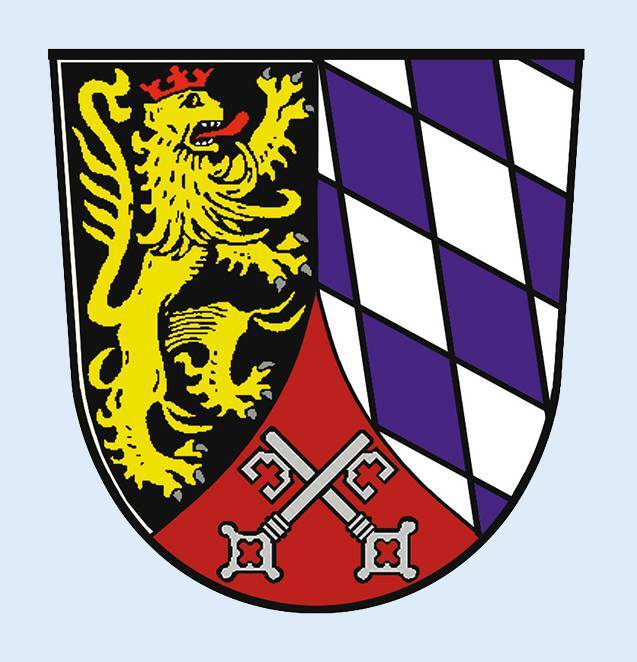
Das Wappen der modernen Oberpfalz als einem von sieben bayerischen Regierungsbezirken spiegelt die gewählten historischen Schauplätze und Protagonisten im Text wider: neben den weiß-blauen Rauten der Wittelsbacher (hinten) der rot-bewehrte und gekrönte Pfälzer Löwe und in der Spitze die silbernen gekreuzten Schlüssel aus dem Stadtwappen der Hauptstadt Regensburg. (Quelle: Wikipedia, gemeinfrei)

**Abb. 2:**
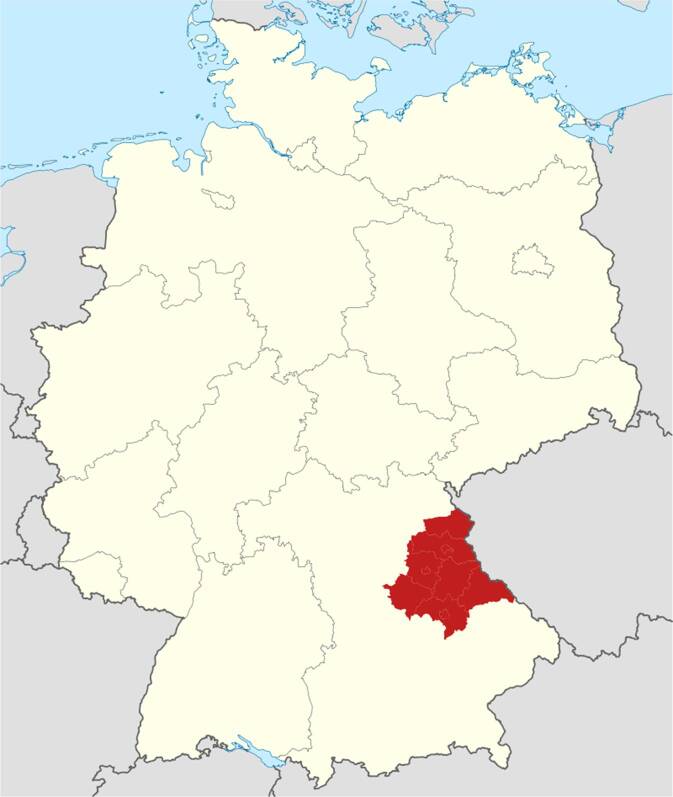
Lage der Oberpfalz (*rot unterlegt*) als an die Tschechische Republik grenzender bayerischer Regierungsbezirk in der Bundesrepublik Deutschland, in Lage und Umfang weitgehend übereinstimmend mit den historischen Regionen „Nordgau“ (8. bis 13. Jahrhundert) und „Obere Pfalz“ (ab dem 14. Jahrhundert). (Quelle: Wikipedia, gemeinfrei)

Als einer der sieben bayerischen Regierungsbezirke existiert die Oberpfalz (Abb. 1) räumlich und nomenklatorisch in der aktuellen Verfassung seit 1837 als dritte kommunale Ebene [1]. Abgesehen von kleineren Gebietsveränderungen, etwa im Rahmen der Gebietsreform von 1972, ist die Oberpfalz mit einer Fläche von knapp 10.000 km^2^ zwar etwa 3,5-mal so groß wie das deutsche Bundesland Saarland, aber mit lediglich 1,11 Mio. Einwohnern deutlich dünner besiedelt als die meisten anderen Regionen der Bundesrepublik (Abb. 2). So ist die Oberpfalz der deutsche Regierungsbezirk mit der geringsten Bevölkerungsdichte (115 Einwohner/km^2^). Dabei existieren auch innerhalb der Oberpfalz große Unterschiede: stetiges Bevölkerungswachstum ist in den städtischen Zentren, allen voran der Hauptstadt Regensburg, zu konstatieren, wo die Bevölkerungsdichte bei 1883 Einwohnern/km^2^ liegt. Insbesondere in den grenznahen Gebieten müssen die Gemeinden aber seit Jahrzehnten einen schmerzhaften Aderlass an menschlichen Ressourcen verkraften, was auch die medizinische Versorgung zunehmend vor Probleme stellt [2].

**Abb. 3:**
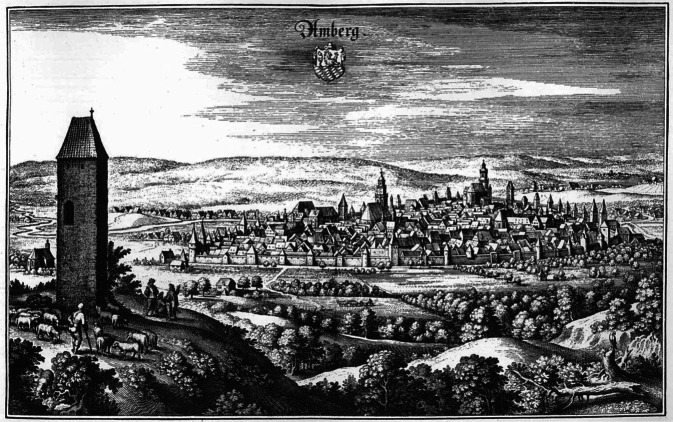
Die Stadt Amberg im Westen der heutigen Oberpfalz war wohl bereits im zweiten nachchristlichen Jahrhundert markomannisch besiedelt und bildete historisch in vielerlei Hinsicht einen Antipoden zur Freien Reichsstadt Regensburg. Der Kupferstich von Matthias Merian (1632) zeigt die Stadt auf dem Höhepunkt ihrer Bedeutung als kurpfälzische Residenzstadt und Hauptstadt der Oberen Pfalz. (Quelle: Herzogin Anna Amalia Bibliothek, gemeinfrei)

Bis zur Bildung des nachnapoleonischen Königreichs Bayern im Rahmen des Wiener Kongresses im Jahre 1815 bestand wie so viele ländliche Regionen in Mitteleuropa auch die heutige Oberpfalz aus einem wahren Flickenteppich reichsunmittelbarer Herrschaftsgebiete [3]. Zur Erleichterung des Verständnisses der weiteren Ausführungen sei hier lediglich auf die zwei seit dem Mittelalter identitätsbildenden Gebietseinheiten, in der längsten Zeit durchaus antipodisch zu verstehen, verwiesen: die historische „Obere Pfalz“ als Teilherzogtum der pfälzischen Linie der Wittelsbacher mit seiner Residenzstadt Amberg (Abb. 3) auf der einen und das seit der Neuordnung Anfang des 19. Jahrhunderts als Hauptstadt der Oberpfalz fungierende Regensburg als römische Gründung, erste bairische Hauptstadt im frühen Mittelalter und von 1245 bis 1810 Freie Reichsstadt auf der anderen Seite [4].

## Die Stadt und das Land – Entwicklungslinien von Regensburg und der Oberpfalz

**Abb. 4:**
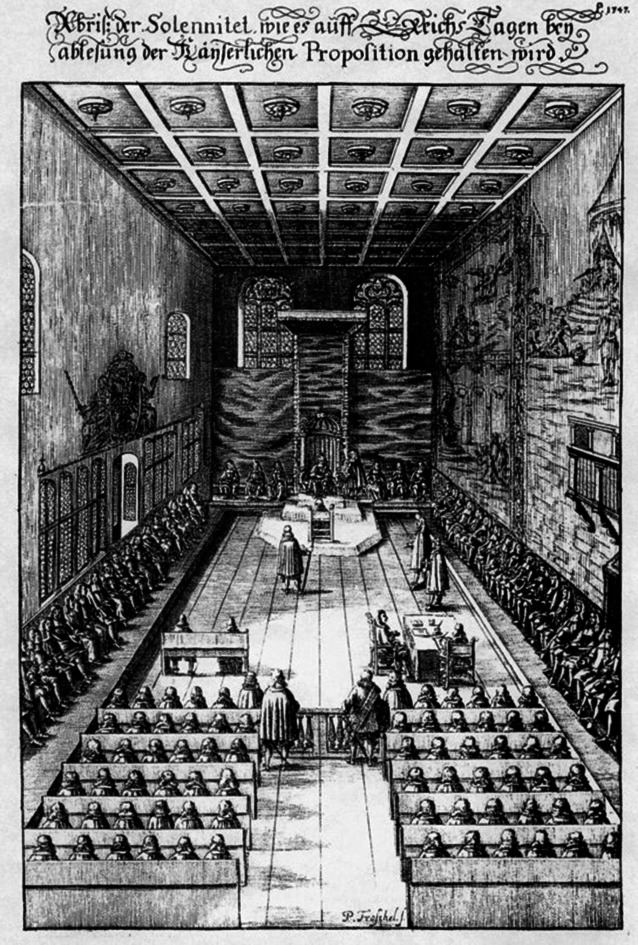
Blick in den Historischen Rathaussaal Regensburg (Peter Troschel, 1675), in dem sich ab 1663 bis zum Ende des Heiligen Römischen Reiches der Immerwährende Reichstag traf. Nach dem Niedergang als hochmittelalterliche Handelsmetropole war dies die Epoche von Regensburgs größter politischer Bedeutung. Hinter dem Kaiser an der Stirnseite nahmen die Kurfürsten Platz, links die weltlichen, rechts die kirchlichen Reichsfürsten, auf den Bänken im Vordergrund die Vertreter der Reichsstädte, davor die Sekretäre und Schreiber. (Quelle: Theatrum Europaeum, gemeinfrei)

Regensburg, die mit heute etwa 152.000 Einwohnern größte Stadt entlang der Donau auf deutschem Gebiet, wurde im Jahre 179 unserer Zeitrechnung von Roms Kaiser Marc Aurel (121–180) als Legionslager „castra regina“ gegründet, galt später als eine wesentliche Keimzelle der nachrömischen, vornehmlich bajuwarischen Staatswerdung im 6. Jahrhundert und dominierte den süddeutschen Raum bis ins Hochmittelalter als internationale Handelsmetropole. Seit 1245 Freie Reichsstadt und ab 1663 bis zum Ende des Heiligen Römischen Reichs Deutscher Nation im Jahre 1803 Sitz des Immerwährenden Reichstags (Abb. 4) als dem ersten „Parlament“ auf dem Europäischen Festland bildete Regensburg, das erst 1810 wieder Teil Bayerns wurde, in der Geschichte noch weit mehr als heute einen Kontrast zum rural geprägten, nördlicheren Raum der Oberpfalz [5]. Bis in die Zeit Karls des Großen (747–814) bildete diese später als „Nordgau“ bezeichnete Pufferzone den östlichen Vorposten der Territorial- und Sprachgrenze des Frankenreichs diesseits des Böhmer-Waldes [6]. Dabei war die Bevölkerung dieses Landstrichs im Ursprung durchaus multiethnisch zu bezeichnen wie zahlreiche aus der slawischen Sprache herrührende Ortsnamen noch heute bezeugen [7]. Der wechselseitige Einfluss der Nachbarn sollte immer wieder relevant werden, etwa im 14. Jahrhundert, als Kaiser Karl IV. (1316–1378) durch Erwerbungen und Pfandnahmen aus örtlichen Herrschaften eine Landzunge durch die heutige mittlere Oberpfalz hin zu seiner Kaiserstadt Nürnberg begründete. Das sog. „Neuböhmen“ hatte aber nur kurze Zeit Bestand, wurde bald nach des Kaisers Tod wieder in den Besitz der Wittelsbacher rückübertragen [8]. Es war jedoch nicht der bairische Familienzweig, der dieses Gebiet nach dem Vertrag von Pavia 1329 im Wesentlichen beeinflussen sollte, sondern der rheinische oder pfälzische, woraus sich auch die nach der Topographie hergeleitete Bezeichnung „Obere Pfalz“ herleitet [8]. Im Gegensatz zu den durch weitere Teilungen im Spätmittelalter und der frühen Neuzeit verzwergten bairischen Teilreiche der Wittelsbacher war der (Ober‑)Pfälzer Herzog bald einer der sieben wichtigsten Fürsten des Reiches und regierte bis zum Ende des alten Reichs auch 200 Jahre im Rheinland als Herzog von Jülich-Berg in vergleichsweise mondänen Verhältnissen [5]. Dies konnte jedoch die zunehmend ärmlichen Bedingungen in der Oberpfalz kaum abmildern, insbesondere nachdem im Laufe des 17. Jahrhunderts die Hochzeit des Erzabbaus und der Eisenerzeugung im einstigen „Ruhrgebiet des Mittelalters“ langsam zum Erliegen gekommen war [9]. Durch Hungersnöte, zahllose Kriege und nicht zuletzt auch Epidemien wurde der Landbevölkerung stark zugesetzt [10]. Das sollte noch lange so bleiben, auch als der letzte überlebende Wittelsbacher Hauptzweig, die Pfalz-Sulzbacher aus der westlichen Oberpfalz, mit Herzog Karl Theodor (1724–1799) im Jahre 1777 erstmals wieder alle Wittelsbacher Gebiete im Kurfürstentum Bayern vereinte [11].

## Eine spätrömische Annäherung an die ersten Regensburger Ärzte

**Abb. 5:**
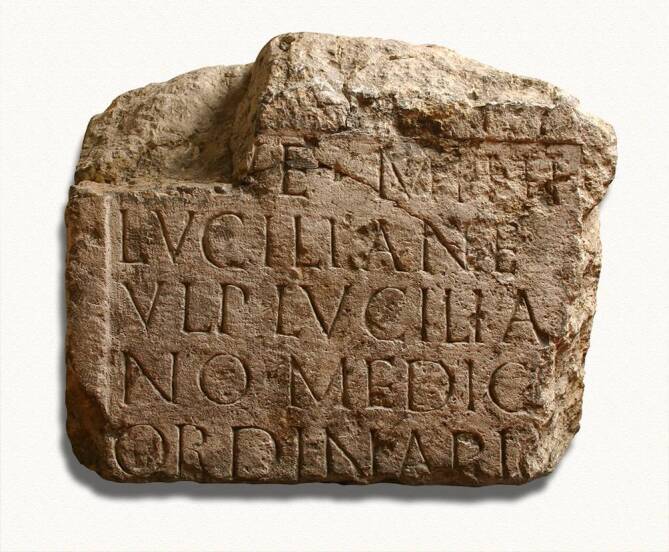
Epitaph des Ulpius Lucilia, der als Militärarzt der 3. Italischen Legion der erste namentlich bekannte Regensburger Mediziner war. (Foto: Ortolf Harl, Bearbeitung ArcTron; Inventarnummer MSR Lap. 31 des Historischen Museums Regensburg)

**Abb. 6:**
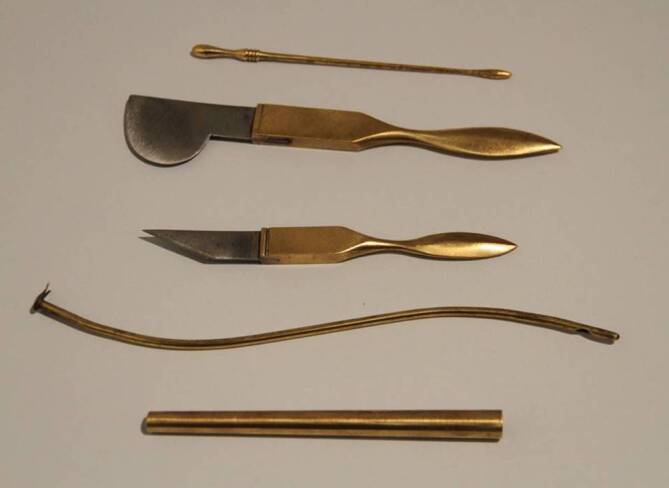
Repliken des britischen Spezialisten Steve Wagstaff von römischen Originalen aus der Zeit Galens von Pergamon, wie sie auch bei Grabungen im einstigen Legionslager „castra regina“ gefunden wurden: von oben Bougierungsstift, Operationsskalpelle und Harnröhrenkatheter. (Quelle: Dr. Christian Koepfer, Augsburg)

Mit dieser vorerst letzten allgemeinhistorischen Vorbemerkung kommen wir langsam zur Hauptthematik dieses Beitrags zurück und begeben uns an die Quellen des Medizinwesens in der vorgestellten Region. Wir wollen dabei nicht bei „Adam und Eva“ anfangen – oder übertragen auf die Geschichtsdidaktik, mit den zahllosen Kulturen, deren Präsenz durchgehend von der Jungsteinzeit bis zu den Kelten archäologisch an der Donauspitze nachgewiesen sind – ein Blick in die Epoche, als das Römische Weltreich auch an der Donau herrschte und dort zum ersten Mal auf deutschem Gebiet so etwas wie ein frühes Staatswesen bildete, soll des geschichtlichen Rückgriffs genügen. Diese Zivilisation wirkte in der im Interesse stehenden Region freilich in erster Linie in dem gegen Ende des 2. Jahrhunderts errichteten Grenzkastell „castra regina“, nur indirekt in den nördlich der Donau gelegenen Raum, der erst in deutlich späterer Zeit Indikatoren eines vergleichbaren Organisationswesens aufweisen sollte. Hier zumindest kann aber davon ausgegangen werden, dass nicht alles, aber doch sehr vieles, was das Leben in den Metropolen des Römischen Reiches so sehr von dem im „freien Germanien“ unterschied, in „castra regina“ vorgehalten wurde. Dazu gehörte insbesondere die medizinische Versorgung. Ein archäologischer Grabungsfund im Regensburg des Jahres 1856 brachte denn auch den Stein gewordenen Beweis zu Tage: Ein Epitaph aus dem 3. Jahrhundert (Abb. 5) erinnert an den ersten namentlich bekannten Mediziner der Oberpfalz, einen gewissen Ulpius Lucilia, der wohl als Militärarzt der 3. Italischen Legion wirkte [12]. Bei weiteren archäologischen Grabungen wurden auch Instrumente zu Tage gefördert, die Ulpius Lucilia und seine Kollegen in der Spätantike anwendeten. Es darf davon ausgegangen werden, dass sich im zentralistisch aufgebauten Römischen Reich die medizinische Tätigkeit in den Provinzen nicht wesentlich von der in den Metropolen unterschied (Abb. 6). Damit dürfte das Repertoire der römischen Chirurgie auch im spätantiken Regensburg praktiziert worden sein, man denke etwa – um auf dem Feld der Behandlung urogenitaler Erkrankungen zu bleiben – an den heute als Celsischer Schnitt bekannten Eingriff zur Entfernung von Harnblasensteinen [13]. Ähnlich wird es sich, wie Funde belegen, mit Katheterisierungen verhalten haben [14].

Im Laufe des 5. Jahrhunderts war mit dem fortschreitenden Druck auf den Donau-Limes, was bereits im dritten und vierten Jahrhundert immer wieder zu Plünderungen „castra reginas“ durch Alamannen und Juthungen (letztere am ehesten von nördlich der Donau anrückenden germanisch-stämmigen Gruppen und Grüppchen abstammend) geführt hatte, das einstige Hauptquartier der 3. Italischen Legion verlassen worden. Freilich hatte sich längst um das Kastell eine Zivilstadt entwickelt, die auch nach dem Abzug der römischen Armee fortexistierte. Zu den seit Generationen nach ihrer Dienstzeit in der Stadt verbliebenen Veteranen der anfangs in Oberitalien ausgehobenen, später aus neueren Untersuchungen aber auch aus orientalischen Provinzen stammenden Legion kamen Bevölkerungsgruppen, die auch im Zuge der Völkerwanderung in der Stadt verblieben waren [15].

## Zwischen Ars liberales und mechanicae – zur Rollensuche der mittelalterlichen Medizin in Regensburger Klöstern und Stiften

**Abb. 7:**
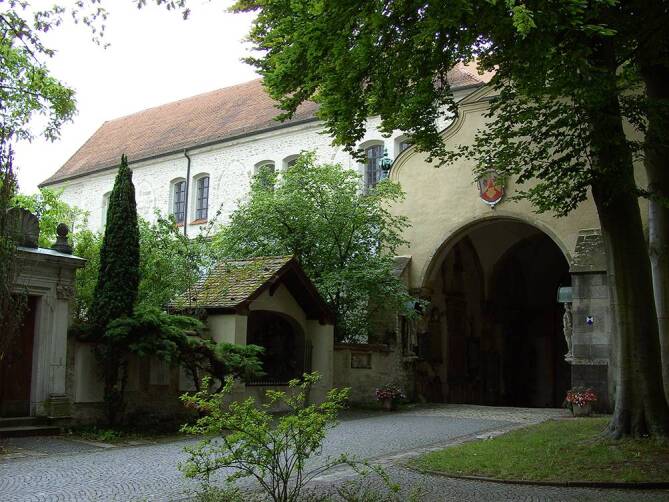
Atrium des Klosters St. Emmeram aus dem 8. Jahrhundert, eine geistliche und geistige Wiege Bayerns. Der Heilige Kaiser Heinrich II. erhielt dort Ende des 10. Jahrhunderts seine Ausbildung zum Herrscher des Heiligen Römischen Reichs Deutscher Nation, neben dem Geschichtsschreiber Johannes Aventinus (1477–1534) wurde hier 1673 auch der „fliegende Steinschneider“ Charles Bernoin beigesetzt. (Quelle: Forum Verlag, Regensburg)

Waren die ersten Zeugnisse uromedizinischen Handels in der späteren Oberpfalz also dem römischen Militärwesen zu verdanken gewesen, so hat die katholische Kirche im Mittelalter das, was wir heute als Gesundheitswesen bezeichnen würden, begründet und geprägt. Zahllose Funde in Ausgrabungen der letzten Jahrzehnte belegen, dass die Stadt Regensburg, von der nachrömischen, aber keineswegs unromanischen Bewohnerschaft bald „Radaspona“ genannt, durchgehend besiedelt blieb. Die Sprachwissenschaft geht so weit zu vermuten, dass es sich bei der Namensschöpfung nicht unbedingt um eine Neubildung handelt, sondern das keltische, also vorrömische „Rataspona“ durchaus über die Jahrhunderte hindurch erhalten wurde [16]. Im Schrifttum bleiben die Entwicklungen vom 5. bis 8. Jahrhundert aber selbst für die „metropolis“ sehr im Dunkeln. Erst Arbeo (723–784), der Bischof von Freising und wohl erste deutschsprachige Geschichtsschreiber hält um das Jahr 770 fest: „Die Stadt […] war uneinnehmbar, aus Quadern erbaut, mit hochragenden Türmen, und mit Brunnen reichlich versehen; im Norden bespült sie die Donau, die in geradem Lauf gen Osten strömt.“ Da ging das erste bairische Stammesherzogtum bereits seinem Untergang entgegen, 788 verleibte es Karl der Große in sein Frankenreich ein. Der bei seinem Vetter in Ungnade gefallene Herzog Tassilo III. (741–796), beide waren Enkel des Karl Martell (gestorben 741) gewesen, hatte kurz zuvor das Stift Niedermünster zu Regensburg gegründet. Dieses Damenstift war bald eines der einflussreichsten seiner Art im deutschsprachigen Raum [17]. Bereits zwei Generationen zuvor war das Benediktinerkloster St. Emmeram gegründet worden, benannt nach einem der heiligen Wanderbischöfe des ausgehenden 7. Jahrhunderts, die die römisch-katholische Lehre zum bajuwarischen Adel brachten, der zwar schon christlich war, aber zumeist dem arianischen Bekenntnis angehangen war. Von besagtem Kloster St. Emmeram, das im selben Jahr 739 wie die noch heute bestehenden altbairischen Diözesen, darunter auch Regensburg, entstand, wurde nicht nur der Nordgau, sondern später auch die angrenzenden böhmischen Gebiete christianisiert (Abb. 7).

Ist ja recht interessant, möchte der ein oder andere Leser jetzt sagen, doch, was hat das mit Urologie zu tun? Eine ganze Menge: dass im Atrium des Klosters, freilich war dies über 900 Jahre nach dessen Gründung, 1673 der „fliegende“ Steinschneider Charles Bernoin (1615–1673) seine letzte Ruhestätte fand, ist dabei natürlich nur eine Randnotiz. Wie jedes große Kloster des Frühen und Hohen Mittelalters hatte man sich auch in St. Emmeram aber den Wissenschaften verschrieben, wobei sich die Medizin in jener Epoche erst auf den Weg dazu machte, als solche Anerkennung zu finden. Noch im 12. Jahrhundert zählte Hugo von St. Victor (1097–1141), ein wohl aus dem Harz stammender Aristokrat, der später in Frankreich als Theologe und Philosoph Bekanntheit erlangte, die Heilkunst noch zu den Artes mechanicae, die im Gegensatz zu den Artes Liberales (das sind zur Erinnerung Grammatik, Dialektik, Rhetorik, Arithmetik, Geometrie, Musik und Astronomie) nicht der geistigen Erbauung, sondern der Lebenserhaltung (zumindest der diese Künste Ausführenden) dienten [18]. Ärzte standen also damals und noch lange Zeit später – mancher wird sagen, dies ist, zumindest bestimmte Aspekte der Medizin betreffend, bis zum heutigen Tage so – auf einer Stufe mit Schmieden, Tuchwebern, Soldaten oder Händlern, weniger mit Wissenschaftlern, wie sie vor der Gründungswelle von Universitäten im Heiligen Römischen Reich Deutscher Nation ab dem 14. Jahrhundert in den Klöstern ausgebildet wurden und die damit im Unterschied zu den Handwerkern (mechanicae) in der Regel keinem Broterwerb nachgehen mussten.

## Ein Regensburger Vorort-Heiliger und sein kaiserlicher Beitrag zur Urologie

Bereits an der Schwelle zum zweiten nachchristlichen Jahrtausend erhielt übrigens auch der bairische Herzogssohn Heinrich seine schulische Ausbildung im schon damals hochangesehenen Kloster St. Emmeram. Unter dem später heiliggesprochenen Bischof und Abt Wolfgang (924–994) und dem Seligen Ramwold (gestorben um das Jahr 1000), die weit über die Grenzen des damaligen Bayerns hinaus wirkten, wurde dem Großneffen Kaiser Otto I. (912–973) das geistliche Rüstzeug für seine spätere Herrschaft auf der einen Seite und sein heiligmäßiges Leben auf der anderen Seite mitgegeben [15].

**Abb. 8:**
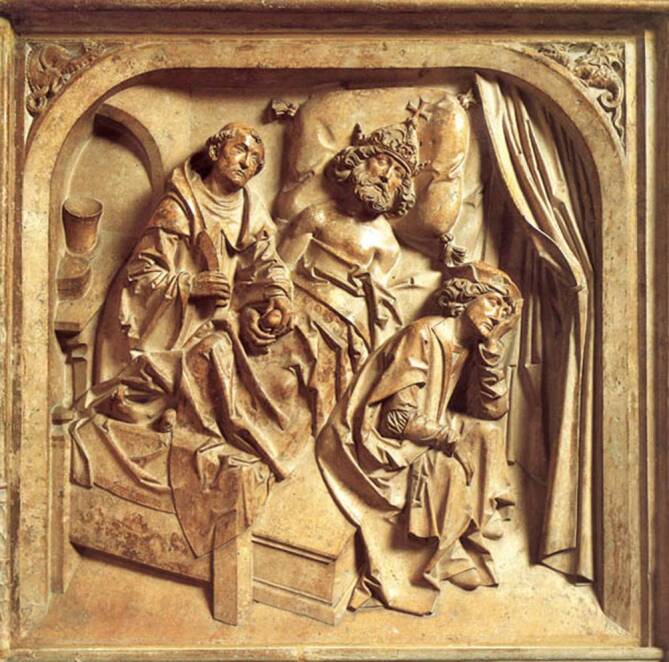
Ausschnitt aus dem Epitaph Tilmann Riemenschneiders (vollendet 1513) für das Kaiserpaar Heinrich und Kunigunde im Bamberger Dom, das die „Steinheilung“ des Kaisers vom Blasenstein, die sich zu Beginn des 11. Jahrhunderts im Kloster Montecassino zugetragen haben soll, darstellt. (Quelle: Historisches Lexikon Bayerns, Foto: Toni Schneiders)

Warum der Autor von den vielen hochadeligen Herren, die dort im Hochmittelalter gebildet wurden, ausgerechnet Heinrich herausstellt? Das mag zum einen daran liegen, dass der spätere Heilige der römisch-katholischen Kirche wohl (da bleibt aus dieser Epoche selbst bei herausragenden Persönlichkeiten immer ein Restzweifel) im Regensburger Vorort Abbach geboren wurde und später zum römisch-deutschen Kaiser Heinrich II. (geboren wohl 973–1024) gewählt wurde. Ganz sicher ist man sich insbesondere beim Geburtsjahr nicht, weshalb man im zwischenzeitlich zum Bad erhobenen Abbach auch lieber 2024 des 1000. Todestages gedenken möchte: der 13. Juli als Todestag und die ottonische Kaiserpfalz Grona bei Göttingen als Todesort sind nämlich historisch verbürgt. Zum anderen, weil hier auch die „Urologie“ wieder ins Spiel kommt: So litt der Begründer des Bistums Bamberg nachweislich ab dem Jahre 1007 unter rezidivierenden Harnblasensteinen, was 1024 auch zu seinem vorzeitigen Tode geführt haben soll [19]. Gänzlich in den Bereich der Mythen ist natürlich die Legende zu verweisen, wonach der Heilige Benedikt von Nursia persönlich den Kaiser Jahre zuvor an diesem Leiden behandelt haben soll, wie es der renaissancezeitliche Meister Tilmann Riemenschneider (1460–1531) am Epitaph des Kaisergrabes in Bamberg kunstvoll der Nachwelt hinterließ [20]. Die künstlerische Freiheit erlaubte es, dass dieser Zusammenhang hergestellt werden durfte – obwohl Benedikt (480–547) bei Heinrichs Besuch auf dem Montecassino bereits fast 500 Jahre tot war (Abb. 8). Durchaus in ernsthafter Diskussion steht dagegen die Beschreibung, dass der Kaiser bei besagtem Besuch tatsächlich am Steinleiden operativ behandelt worden sein könnte. Das große medizinische Wissen der Mönche zu dieser Zeit und insbesondere in dieser durchaus auch arabisch-medizinischen Einflüssen unterworfenen Region Latium in Mittelitalien mag dies tatsächlich ermöglicht haben [21, 22].

Über dieses biographische Kuriosum hinaus hat das Kloster St. Emmeram in seinem reichen hochmittelalterlichen Buchschatz aber mit „De viribus herbarum“ immerhin auch zumindest ein Werk bewahrt, das nicht nur für die althochdeutsche Sprachwerdung Bedeutung erlangte, sondern das mittelalterliche Standardwerk der Kräuterheilkunde darstellte. Es handelt sich um eine im 13. Jahrhundert wohl in St. Emmeram übersetzte Fassung der in lateinischen Hexametern gehaltenen Ursprungsversion aus der Mitte des 11. Jahrhunderts [23]. Der Nachweis operativer Eingriffe, wie sie der Heinrichslegende in Montecassino zugrunde lag, fehlen für die Regensburger und Oberpfälzer Klöster allerdings.

## Wie vom mittelalterlichen Adelssterben auch das Hospiz- und Medizinalwesen in Regensburg und dem Nordgau profitierte

Sicherlich haben andere Schriften, insbesondere theologische, astronomische oder juristische, in den Regensburger Klöstern größere Bedeutung erlangt, dazu entstand hier ein reicher Schatz an hebräischen Werken – die Stadt an der Donau beherbergt die älteste israelitische Kultusgemeinde Bayerns und war im Hochmittelalter neben den SchUM-Städten am Rhein ein Zentrum des europäischen Chasside Aschkenas – gleichwohl dürfte die praktische Anwendung der in medizinischen Schriften erwähnten Rezepturen den üblichen großen Raum auch dort eingenommen haben [24, 25]. Erst recht in den ab dem Jahre 1100 gegründeten ländlich geprägten Klöstern des Nordgaus, die im Wesentlichen erst die Urbarmachung und Besiedelung dieses weitgehend noch Urwaldgebiet entsprechenden Raumes, den wir heute Oberpfalz nennen, ermöglichten. Die Klöster Kastl im Westen, Waldsassen im Norden und Walderbach bzw. Reichenbach im Osten sind hier insbesondere zu nennen. Als Gründer fast aller dieser Klöster ging jeweils Markgraf Diepold III. (1075–1146) ein, Schwiegervater Kaiser Friedrich Barbarossas (1122–1190) und wohl einer der bedeutendsten Vertreter seines Geschlechts der Rapatonen und seiner ganzen Klasse im ausgehenden 11. und beginnenden 12. Jahrhundert [26].

**Abb. 9:**
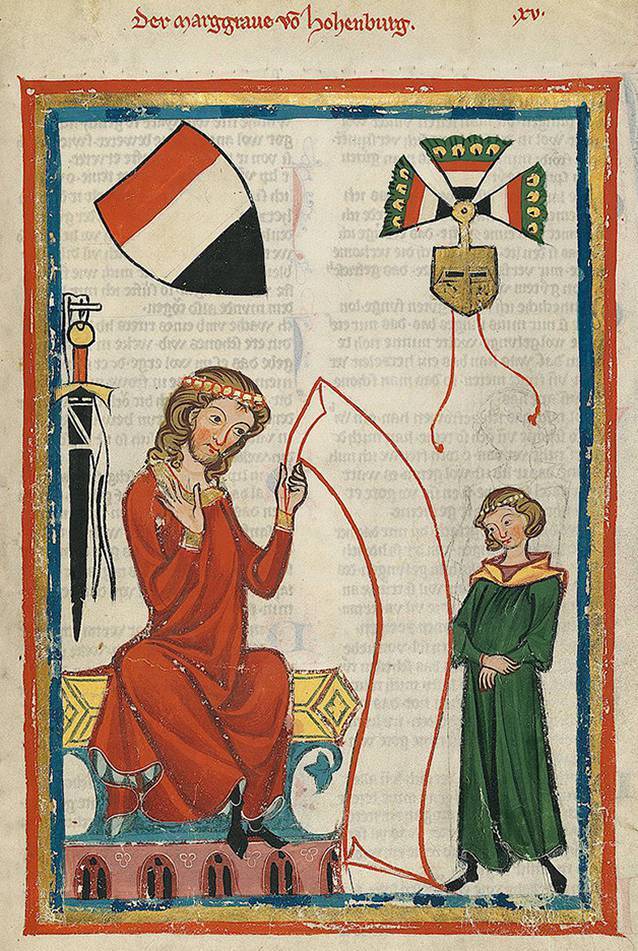
Darstellung des Markgrafen Berthold von Vohburg-Hohenburg (1215–1257), erfolgreicher Politiker und Minnesänger von europäischer Bedeutung aus dem Nordgau auf einer historischen Darstellung im Codex Manesse, erstellt etwa 50 Jahre nach dessen gewaltsamen Tod Anfang des 14. Jahrhunderts. (Quelle: Codex Manesse, gemeinfrei)

Die Familie der Rapatonen könnte pars pro toto für Aufstieg und Fall einer Uradelsfamilie im östlichen Bayern stehen: erstmals im Rahmen der Schlacht auf dem Lechfeld (955) aufgetreten wuchs der Einfluss der auch Diepoldinger genannten Adelsfamilie im 11. und 12. Jahrhundert stark an, um im 13. Jahrhundert ein jähes Ende zu finden. Während ein großer Teil der damaligen Adelsgeschlechter auf oder an einem der Kreuzzüge zugrunde ging, war es beim letzten Markgrafen von Vohburg, dem wichtigsten Adelsgeschlecht seiner Zeit auf dem Nordgau, die Verstrickung in andere Belange der Politik. Ehe der letzte Markgraf Berthold, zwischenzeitlich Regent des Königreichs Sizilien, in die Mühlen der Weltgeschichte geriet (nachzulesen in Dante Alighieris „Commedia Divina“) betätigte er sich immerhin noch eifrig als Minnesänger, blieb aber ebenso kinderlos wie der letzte Vertreter des zweiten wichtigen Geschlechts auf dem Nordgau, Berengar II. von Sulzbach (gestorben 1167; Abb. 9). Dessen Tante Berta, geboren 1110 im heutigen Sulzbach-Rosenberg, war die einzige deutsche Prinzessin (ihr Schwager war der römisch-deutsche König Konrad III.), die jemals als Kaiserin des Byzantinischen Reiches auf dem Thron in Konstantinopel saß. Sowohl Berta als auch Berthold (1215–1257) lebten in einem Umfeld, in dem auch die chirurgische Heilkunst durch stärkeren Einfluss der byzantinisch-arabischen Medizin bereits einen ganz anderen Stellenwert genoss als in ihrer Heimat [27].

Auch Konrad von Frontenhausen (1170–1226), von 1204–1226 Bischof von Regensburg, um wieder in die Metropole der Oberpfalz zurückzukommen, war der Letzte seines Namens. Die Familie der Frontenhauser starb mit dem hohen geistlichen, aber auch weltlichen Herrn (1205–1208 Reichskanzler des römisch-deutschen Königs Philipp) im Mannesstamm wie so viele bairische Adelsfamilien in dieser Zeit aus [28]. Für das Hospiz- und im weitesten Sinne auch Medizinalwesen der Stadt war dies aber ein großes Glück, das bis zum heutigen Tage andauert. Denn sein ererbtes Vermögen von 7000 Pfund Regensburger Pfennigen, die der Bischof noch zu Lebzeiten dem von ihm aus der Stadt an das nördliche Donauufer verlagerten Johannesspital mit Ursprung wohl im 10. Jahrhundert vermachte, begründeten die Bedeutung des bis zum heutigen Tage bestehenden Katharinen-Spitals [4, 29].

**Abb. 10:**
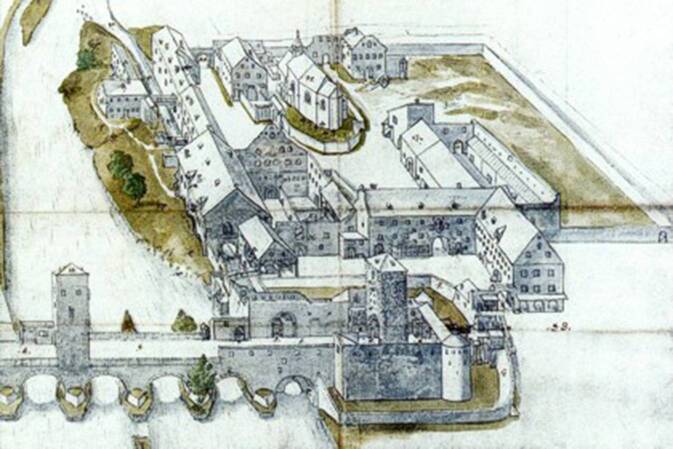
Das St. Katharinenspital auf einer späteren Darstellung aus dem Jahre 1656, auf dem die exklavische Lage des Areals, wohin das Spital 1226 aus der Stadt auf die Nordseite der Donau verlegt worden war, deutlich wird. Die Ursprünge als Johannesspital gehen wohl auf das 10. Jahrhundert zurück. (Quelle: Bayerisches Hauptstaatsarchiv, gemeinfrei)

Die Kreuzzüge des Hohen Mittelalters waren aber, drücken wir es ein wenig euphimistisch aus, nicht nur indirekt verantwortlich für so manche Erbschaft, die Klöstern und frühen Hospizeinrichtungen zu Nutz und Segen gereichten. Auch ganz direkt machten sich insbesondere die Folgen des dritten Kreuzzuges (1189–1192, 1197/1198) in Regensburg bemerkbar. Das 1190 vor Akkon von bremischen und lübeckischen Kreuzfahrern gegründete „St. Marien-Hospital der Deutschen zu Jerusalem“ konzentrierte sich – inzwischen war aus den ursprünglich und ausschließlich der Krankenpflege verschriebenen Gemeinschaft der bald unter päpstlichem Generalprivileg stehende Deutsche Orden geworden – mit Beginn des 13. Jahrhunderts auf Tätigkeiten in Zentraleuropa. Mit zahlreichen Schenkungen bedacht, so auch von Baiern-Herzog Ludwig I. (1173–1231), entstand in Regensburg ab dem Jahr 1210 die erste Niederlassung des Deutschen Ordens im heutigen Bayern [30]. Wie beim Beispiel Katharinen-Spital (in einer Exklave der alten Reichsstadt gelegen, jedoch mitten in der einstmals bairischen Stadt Stadtamhof, Abb. 10) existiert die Alten- und Krankenpflege bis heute im Deutschordenshaus in der Altstadt von Regensburg fort.

## Beginn des spätmittelalterlichen Spitalwesens in der wittelsbachischen Oberen Pfalz

Mit dem Deutschordenshaus St. Ägid lassen wir das Gebiet der Metropole einerseits und die monastisch-orientierte Krankenbehandlung andererseits hinter uns. Denn auch im „Nordgau“ machte sich das Rodungs- und Städtebauprogramm der sich nach der Belehnung mit dem Herzogtum Baiern in Wittelsbacher umbenannten Grafen von Scheyern eindrucksvoll bemerkbar. Bis etwa 1270 hatten die Wittelsbacher, von kirchlichen Besitzungen und der Landgrafschaft Leuchtenberg abgesehen, weite Teile der späteren Oberpfalz, die freilich erst etwa 60 Jahre später im Rahmen des Hausvertrags von Pavia eine territoriale Begrifflichkeit werden sollte, unter ihre Herrschaft gebracht [31]. Aus hegemonialem Impetus heraus, gefördert von einem sich mehr und mehr emanzipierenden Bürgertum in den Marktflecken und Städtchen, bildeten sich nach dem Vorbild kirchlicher Spitäler zahlreiche Institutionen heraus, die das gesamte Spektrum der Wohlfahrtspflege vereinten: Kranken- und Altenpflege, Versorgung von Weisen und Fremden. Sie bilden damit den gemeinsamen historischen Ursprung nicht nur des Hotelwesens, von Kinder- und Altenheimen, sondern auch des Krankenhauses moderner Prägung. Zu nennen sind hier aus dieser Zeit in der Oberen Pfalz etwa Neumarkt (1240), Cham (vor 1285), Kastl (vor 1302), Sulzbach (vor 1320), Freistadt (1305), Weiden (1382), Auerbach (1384), Neunburg vorm Wald (1398) und als prominenteste Gründung das 1317 vom römisch-deutschen König Ludwig dem Bayern gestiftete Bürgerspital zu Amberg [10].

Der erste Wittelsbacher Kaiser starb 1347 und musste das im Folgejahr über Europa anbrechende Zeitalter der großen Seuchen nicht mehr miterleben. Aber auch hierauf fanden die Alten, auch in der Oberpfalz, ihre Antworten.
